# Improvement of cervical spine mobility and stance stability by wearing a custom-made mandibular splint in male recreational athletes

**DOI:** 10.1371/journal.pone.0278063

**Published:** 2022-12-15

**Authors:** Johanna Herzog, Florian Göttfert, Christian Maurer-Grubinger, Fabian Holzgreve, Gerhard Oremek, David A. Groneberg, Daniela Ohlendorf

**Affiliations:** 1 Dental Practice of Edel & Weiß, Nuremberg, Germany; 2 Institute for Occupational Medicine, Social Medicine and Environment Medicine, Goethe-University Frankfurt, Frankfurt am Main, Germany; University of Catanzaro, ITALY

## Abstract

**Objectives:**

The range of motion (ROM) of the cervical spine and postural stability are important for an economical and motorically adequate adaptation of the body to any situation. Therefore, this study aims to analyze whether these two components of postural and movement control can be influenced by means of a splint in a centric position compared to habitual occlusion.

**Methods:**

38 recreational male athletes volunteered. Cervical spine ROM was recorded using an ultrasound system and the a pressure measuring plate for postural stability (length of center of pressure (CoP) movement, area of CoP). The two dental occlusion conditions employed were the habitual occlusion and wearing a splint in an idealized, condylar position close to the centric position. Level of significance was set at ρ ≤ 0.05.

**Results:**

The cervical spine mobility increased significantly by wearing the splint regarding rotation to the left (+3.9%) and right (+2.7%) and lateral flexion to the left (+4.4%) and right (+6.7%). Wearing the splint reduced the area of sway deflections by about 31.5% in the bipedal stance and by about 2.4% (left) and 28.2% (right) in the unipedal stance. The CoP trace was reduced in the sagittal plane by approximately 8.2% in the right single-leg stance.

**Conclusions:**

The major findings seem to demonstrate that wearing a splint that keeps the jaw close to the centric relation may increase the cervical ROM and may improve balance stability in male recreational athletes. Changing the jaw relation in athletes can possibly aid the release of performance potentials by improving coordination skills.

## Introduction

Coordination is essential for both daily and physical activities. It is defined, amongst other things, as the ability to perform motor responses in a smooth, accurate and controlled manner; this requires optimal intra- and inter-muscular coordination on the one hand and optimal interaction of muscle function at equivalent intensity on the other [1, 2]. A coordinated movement is identifiable when speed, distance, direction, timing and muscular tension are used appropriately. The motor response is supported by other physiological components such as proprioception, vision or the vestibular system and cerebellum [1]. The motor response is also dependent on the flexibility and range of motion (ROM) of the body segments involved [1]. In this way, the body can adapt economically and motorically to any situation with certainty. In this context, the ability to keep one’s balance is also an important component; this is controlled and maintained via postural control. Postural control is supported by the visual system, which, due to the position of the cervical spine, is always directed towards balancing body fluctuations in the upright stance in the best way possible [2].

Well-developed postural control is an important requirement, especially in (competitive) sport, which can be key for sporting success. In order to achieve sporting success, external support options, are used to improve sporting performance, like a nose patch for better ventilation, caffeine or nicotine intake, individually-adapted shoes or insoles for superior economical movement [3] as well as dental approaches through the use of specially produced splints or mouth guards [4–7].

The latter support option has already been scientifically investigated many times in order to gain an understanding of the effects of dental changes in the temporomandibular system (TMS) on various components of the musculoskeletal system [1, 8–26]. The reason why this aspect is the subject of many investigations lies in the neurophysiological interdependence of the cervical spine-mandibular axis, which can be explained theoretically. This includes the interference between the sternocleidomastoid muscles and the mandibular. Attempts have been made to explain this regulatory mechanism in a biomechanical model [27, 28] or to generate direct derivatives by means of experiments [29–31]. The analysis of dental occlusal changes in the TMS on cervical spine mobility [9, 17, 32–34] or postural control [35–42] are two important assessment components in this regard. In this context the teeth of the upper jaw are in contact with those of the lower jaw almost every time the patient swallows [43]. The position of the mandible is determined solely by the position of the upper teeth; this means that the mandible in an incorrect, forced position leads to changes and incorrect loads in the TMS [43]. By intervening in this system with occlusal splints, the occlusion can be changed and, with the help of neurophysiological mechanisms, influence can be exerted on the TMS and its associated structures such as the cervical spine position, adjacent caudally [44] or postural control, further away caudally [43]. Dental interlocking can be influenced by means of individually produced splints on a wide variety of structures located at a distance from the TMS caudally; these can be evaluated by using various measurement methods [45–48].

A multifactorial, musculoskeletal temporomandibular disorders (TMD) etiology may develop from parafunctional habits, such as teeth clenching, local or systemic disease, or from psychosocial problems, such as anxiety [49]. Regarding TMD type, Ferillo et al. [50] found that arthritic and mixed TMD complaints correlate with mild to moderate TMD pain (depending on neck pain intensity), while myogenic TMD are correlated with moderate to severe TMD pain. In this context, pain is often clinically manifested. In this context, the rehabilitative therapy is of great importance. A meta-analysis demonstrates that all of the conservative approaches studied, such as laser therapy, dry needling, physical therapy, percutaneous needle electrolysis, transcutaneous electrical nerve stimulation, acupuncture, and ozone therapy, can significantly reduce pain in patients with muscle-related disorders [51]. An additional possible effective therapy option for pain relief is the combination of radial Extracorporeal Shock Wave Therapy with physiotherapy [49]. Another option in the case of patients with TMD are occlusal splints. When worn for a long time, have also a good postural influence according to Ferrillo et al. [52]. They further summarize that there is a consensus in the scientific literature on the use of rigid occlusal splints with a flat occlusal plane for patients with TMD as a noninvasive therapeutic approach. However, the clinical significance of the effects on postural parameters is considered to be low. Furthermore, they urge for a multidisciplinary approach, as well as a combined diagnostic strategy of stabilometric and kinematic evaluation of the spine. Also, Zhang et al. [53] also concluded in their review that an occlusal splint can be used as a noninvasive treatment approach for patients with TMD, especially for those patients who have signs and symptoms of mandibular motion restriction and pain. Possibilities for splint production include a temporarily fixed position of the occlusion, e.g., in the centric relation or the maximum clenching position [54–56].

This study will now analyze whether similar positive results can be demonstrated in people without TMD. These effects could then possibly also have a positive impact on the movement quality of athletes. Therefore, the influence of an individually produced mandibular splint in an idealized, condylar position close to the centric position on the mobility of the cervical spine and on postural stability in male adults without complaints in the TMJ is to be examined. Since hormonal differences in muscle development between men and women may be a possible influencing factor in the planned investigations, only male participants were permitted to volunteer in this study [57–59]. In addition, according to subjective information, all participants were recreational athletes with a high training volume since athleticism—the extent of which is determined by the respective sport—can positively favor postural control [60–62]. This study design has already been used in a study of male and female healthy adults according to sex differences [63]. Therefore, the following hypotheses were investigated in this study:

1. Wearing a splint in an idealized, condylar position close to the centric position leads to an improvement in the maximum cervical movement range in the frontal and transverse planes but not in the sagittal plane.
2. Wearing a splint in an idealized, condylar position close to the centric position leads to a unipedal reduction in the CoP sway area.
3. There is no difference in the CoP path length while wearing a splint in an idealized, condylar position close to the centric position or standing in habitual occlusion.

## Methods

### Participants

In this study, a total of 38 males aged 19 to 65 years (32.87 ± 11.29 years) volunteered.

*Inclusion criteria* were healthy male athletes between 18–65 years with a training load of at least 2x/week, each session being approximately 30–60 minutes in length, a complete dentition (except the third molars), never have worn an occlusal splint before the measurements and no injuries to the musculoskeletal system (such as bone fractures or muscle strains) and the temporomandibular system within the last 2 years prior to the study. In addition, all participants subjectively confirmed to feel healthy which means that they do not to suffer from peripheral, central vestibular or somatosensory disorders as well as disorders of the vestibular organ. The sports activities most frequently mentioned by the participants were soccer, bobsledding, triathlon, basketball, jogging and CrossFit.

*Exclusion criteria* are an acute TMD, current or chronic injuries of the musculoskeletal system, orthodontic or orthopedic treatment at the time of the examination, use of muscle relaxants, acute infection or genetic muscle diseases. In order to exclude acute TMD, the TMD screening form of the German Society for Functional Diagnostics and Therapy (DGFDT) [64] is used and exclude such participants with pain in the temporomandibular joint, pain in the masticatory muscles, temporomandibular joint noises or clicking, occlusion disorders, or limitations in mouth opening or mouth closing.

All participants were informed of the benefits and risks of the investigation prior to signing an institutionally approved informed consent document to participate in the study. An approved ethics application from the medical faculty of the Goethe University Frankfurt has been submitted for the implementation of the study (ethics vote no. 331/2011).

### Measurements

#### Splint production for the individually custom-made mandibular splint

Initially, impressions of the upper (maxillary) and lower (mandibular) jaws were taken from each subject by using alginate (HS-Ortho Alginate, Henry Schein Dental International, Melville, USA). Subsequently, casts were made from super hard plaster (HS-Superhard Plaster Natur, Henry Schein DENTAL, Langen, Germany) which were then analyzed for articulation purposes. The maxilla was articulated according to the Plane System (Plane Finder according to Udo Plaster^®^, Nuremberg, Germany) and the mandible in centric relation to the maxilla.

The centric bite was recorded after wearing an Aqualazier for at least 20 minutes with the aim to deprogram the neurophysiological system [65–72]. Subsequently, the centric bite registration was executed with the aid of ballistic movements and a custom-made anterior jig (millable, auto-polymerizing plastic; LuxaBite, DMG Chemisch-Pharmazeutische Fabrik GmbH, Hamburg, Germany) and a suitable material for injecting under the teeth (VPS HydroBite, Henry Schein UK, Gillingham, United Kindom). The person uses this jig in the anterior region to perform predefined movements, such as protrusion or laterotrusion, without the help of the dentist and thus determines the bite position.

This determined position of the casts in relation to each other formed the baseline for the individual mandibular splint. An adhesive (Mollosil, Detax, Ettlingen, Germany) was applied to create a bond between the splint and the Odontosil silicone (Shore hardness 50; Henry Schein DENTAL, Langen, Germany) after first reducing the splint by milling. The surface of the splint, made of polymethylmetachrylate (PMMA), was reduced in the milling unit so that an approximately 2mm layer thickness of Odontosil could be applied to the splint base without increasing the registered vertical.

*Cervical spine mobility measurement*. The Zebris CMS 70P (Zebris^®^ Medical GmbH, Isny im Allgäu, Germany) was used to record cervical spine movements on the basis of ultrasound pulses. The sampling frequency of each sound wave was set at 25 Hz. All cervical spine movements were registered via a special head attachment with integrated triple markers of which one functioned as a reference triple marker and was attached to the subject to compensate upper body movements. Inter-rater reliability and validity of this system have been classified as good to excellent [73].

*Balance stability measurement*. The balance stability measurements were conducted by pressure sensors (10240 calibrated capacitive sensors), in an area of 170 cm x 65 cm, which were integrated into the treadmill system (FDM-T) from Zebris^®^ Medical GmbH (Isny im Allgäu, Germany). The measuring frequency used was 300 Hz, whereby the pressure was measured in pascals (Pa; force/area unit). The area of the ellipse and the length of the Center of Pressure (CoP) were recorded. According to the manufacturer’s specifications, the measuring range is between 1-120N/cm2 and the accuracy of the calibrated measuring range (1-120N/cm2) is ±5% of the final value.

### Measuring procedure

All participants were assessed consecutively in two conditions: habitual dental occlusion (HO) and with the splint in an idealized, condylar position close to the centric position (S). A coin toss decided which measurement was carried out first. There was always a period of at least 15 minutes passed between the two measurement conditions. Particular attention was paid that after the splint insertion the measurements were started after 20 minutes in order ensure adaptation to the temporary situation by the TMS.

Several test measurements of cervical spine mobility and balance stability were taken prior to data recording. The procedure for carrying out the investigation with the two measuring systems will now be explained in more detail below.

*Cervical spine mobility*. Firstly, the head attachment and upper body reference markers were fixed. The starting position in the sitting posture was as follows: the angle between the upper and lower legs in an upright upper body position was approximately 90°, the arms or hands were rested evenly on the thighs, the gaze was directed straight ahead at eye level, the inclination of the backrest was 70°, the length of the backrest was 45 cm, the inclination of the seat was 5°, the length of the seat was 40 cm and the height of the seat was 45 cm. The calibration was performed in a neutral position, i.e. in a given sitting position, with the gaze directed straight ahead, fixing at a point at eye level. The bite was executed either in habitual occlusion or with the splint lightly fixed. Five repetitions of each of the maximum movement executions (the manufacturer describes these as the range of motion (ROM)) were noted (see below). Three different directions were measured for each of the five repetitions:

For movement 1, the head was flexed as far as possible to the chest or extended to the neck without interrupting the movement (flexion and extension of the cervical spine in the sagittal plane). Movement 2 recorded the axial rotation in the transversal plane of the head for which the head was maximally rotated to the left side and to the right side. Movement 3 comprised the lateral flexion of the cervical spine, including the alternating sideward movement of the head to the right or left shoulder.

*Balance stability*. Balance stability was measured two-fold: barefoot in the bipedal stance and barefoot in the unipedal stance on the left and right leg, respectively. For the bipedal stance, the feet were placed hip-width apart in a habitual posture and the eyes focused on a point on the wall. The same visual focusing also took place for the unipedal measurements.

An acoustic signal started and stopped each measurement period of 10 seconds.

Evaluation criteria were the "Center of Pressure" path length (mm) (total length of the CoP path movements to the anterior and posterior) and the sway area (mm^2^) (95% confidence ellipse for the mean of the CoP anterior, posterior, medial and lateral coordinates). Of importance, for the CoP, the shorter the distance of the CoP path and the smaller the area, the greater the stance stability becomes [74].

### Statistical analysis

All measurement repetitions were averaged for evaluation. The software program BIAS (Version11.8; Epsilon-Verlag, Darmstadt, Germany) was used for the statistical analysis.

The comparison of all evaluation parameters between the left- and right-handers was performed only descriptively due to the small sample size of left-handers (n = 5).

The data were first tested for normal distribution by using the Kolmogorov-Smirnov adjustment test. As the collected measurement data were not normally distributed, median, 1st and 3rd quartile, the two-sided 95% confidence interval as well as the non-parametric Wilcoxon matched-pairs test was used. Consequently, the data have been subjected to Bonferroni-Holm correction. The significance level was 5%.

## Results

The 38 healthy male participants were between 19–65 years old (32.87 ± 11.29 years). Table 1 includes the distribution of participants’ dominant hand and leg.

**Table 1:** Distribution of participants’ dominant hand and leg.

	Right dominant	Left dominant
Dominant hand	33	5
Dominant leg (preferred kicking limb [75])	24	14

Of the 5 left-handers, only one reported the left leg to be their dominant leg. This is in contrast to the right-handers; 13 of the 33 right-handers indicated a left dominant leg.

Table 2 includes the median, 1st and 3rd quartiles and p-values (after Bonferroni-Holm correction) of the habitual occlusion condition compared to wearing the splint, with respect to the cervical spine evaluation parameters. Rotation to the left and right (p ≤ 0.001 and 0.01, respectively) and lateral flexion to the left and right (p ≤ 0.001) showed significant differences between the two conditions. While the rotation to the left increased from 74.00° to 77.00° due to the splint, the right rotation increased from 73.00° to 77.00°. The lateral comparison (left vs. right) with habitual occlusion (p ≤ 0.001) was just as significantly different as that with the splint (p ≤ 0.01).

**Table 2:** Comparison of the parameters of the cervical spine analysis with median, 1st and 3rd quartiles, left and right limit of the confidence interval and p-value while wearing the splint and for the habitual dental occlusion. Significant p-values after Bonferroni-Holm correction are highlighted in bold. The effect size is classified according to Rosenthal as follows: 0.1 "small effect" = 1; 0.3 "medium effect" = 2; 0.5 "large effect" = 3. This classification is superscripted behind the p-value.

	Median	1st Quartile	3rd Quartile	Confidence interval (left/right limit)	Median	1st Quartile	3rd Quartile	Confidence interval (left/right limit)	p-value
	Splint				Habitual dental occlusion				
ROM flexion (°)	72.00	67.00	77.50	68.00/76.00	69.00	60.00	77.50	62.00/76.00	0.07^1^
ROM extension (°)	58.00	49.00	64.50	51.00/62.00	58.00	51.00	64.50	52.00/62.00	0.15^1^
Rotation left (°)	77.00	73.00	81.50	74.00/80.00	74.00	68.50	80.00	72.00/79.00	**0.001** ^**2**^
Rotation right (°)	75.00	68.50	79.00	73.00/87.00	73.00	66.50	77.00	67.00/84.00	**0.01** ^1^
Lateral flexion left (°)	46.00	36.50	52.00	40.00/50.00	44.00	35.00	50.50	40.00/47.00	**0.001³**
Lateral flexion right (°)	45.00	38.50	49.50	41.00/49.00	42.00	36.00	49.00	39.00/47.00	**0.001³**

Flexion to the left increased from 44.00° to 46.00° and the flexion to the right from 42.00° to 45.00° with the splint. The comparisons between habitual occlusion and wearing the splint regarding ROM flexion (p ≤ 0.07) and extension (p ≤ 0.15) were not significant. The lateral comparisons between the left and right body side per measurement condition (HO or S) were not significantly different for lateral flexion (p ≤ 0.92 and p ≤ 0.70, respectively).

The difference between both conditions concerning left and right rotation was also not significant, as was the case with lateral flexion (p ≤ 0.06 or 0.98, respectively). There is a large effect in terms of lateral flexion to the left and right. Otherwise, it is mainly a small effect.

**Fig 1:**
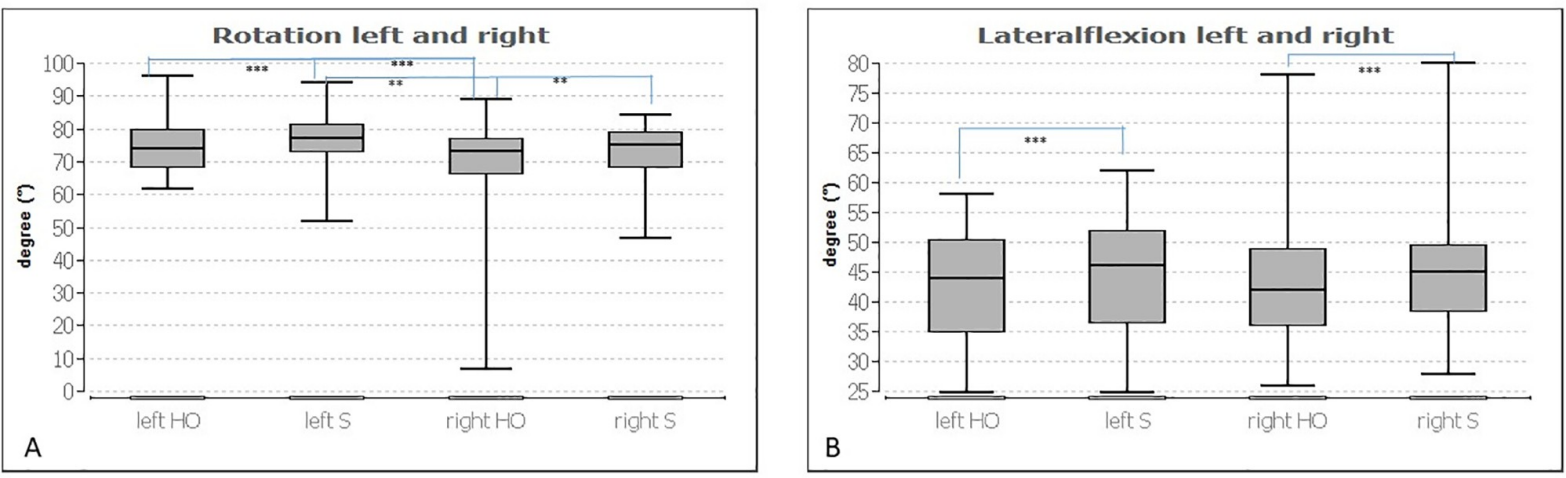
Illustration of A) rotation (left/right) and B) lateral flexion (left/right) with habitual occlusion (HO) and wearing the splint (S). The graphs show box plots with frames representing the 1st and 3rd quartiles and the median demonstrating the thick bar within this box. The upper and lower whiskers illustrate the minimum and maximum. *: p ≤ 0.05, **: p ≤ 0.01, ***: p ≤ 0.001.

Fig 1 illustrates the statistical results for the cervical spine analysis summarized in Table 2.

Table 3 shows the median, the corresponding 1st and 3rd quartiles, left and right limit of the confidence interval and the p-values (Bonferroni-Holm corrected) of all parameters, with and without a splint.

**Table 3:** Comparison of parameters in postural control measurement with median, 1st and 3rd quartiles, left and right limit of the confidence interval and p-value while wearing a splint and for habitual dental occlusion. Significant p-values after Bonferroni-Holm correction are highlighted in bold. The effect size is classified according to Rosenthal as follows: 0.1 "small effect" = 1; 0.3 "medium effect" = 2; 0.5 "large effect" = 3. This classification is superscripted behind the p-value.

	Median	1st Quartile	3rd Quartile	Confidence interval (left/right limit)	Median	1st Quartile	3rd Quartile	Confidence interval (left/right limit)	p-value
	Splint				Habitual dental occlusion				
CoP sway area bipedal (mm^2^)	65.00	45.00	112.00	50.00/73.00	95.00	64.50	168.00	66.00/107.00	**0.01** ^**1**^
CoP sway area left leg (mm^2^)	572.00	419.00	746.50	455.00/613.00	586.00	457.20	943.00	471.00/679.00	0.02^**1**^
CoP sway area right leg (mm^2^)	499.00	419.50	757.00	443.00/699.00	695.00	515.00	938.00	602.00/831.00	**0.001** ^**2**^
CoP path length bipedal (mm)	119.00	81.50	178.50	95.00/148.00	132.00	82.50	156.00	87.00/140.00	0.57^**1**^
CoP path length left leg (mm)	350.00	288.00	400.50	306.00/393.00	338.00	292.50	433.50	315.00/419.00	0.08^**1**^
CoP path length right leg (mm)	320.00	263.50	399.50	276.00/375.00	351.00	267.00	440.50	281.00/381.00	**0.001** ^**1**^

There were significant differences between the two measurement conditions, with and without wearing a splint, for the CoP sway area in the bipedal stance (p ≤ 0.01) and right leg in the unipedal stance (p ≤ 0.001) as well as for the CoP path length in the right leg (p ≤ 0.001). Wearing the splint reduced the CoP area in the bipedal stance (HO: 95.00 mm^2^; S: 65.00 mm^2^) and increased it in the unipedal right stance (right = HO: 695.00 mm^2^; S: 938.00 mm^2^). The CoP path length of the right leg decreased significantly from 351.00 mm to 320.00 mm by wearing the splint (p ≤ 0.001).

The CoP path length in the bipedal stance and for the left leg in the unipedal stance were not significant between the two conditions. The side-by-side comparison per measurement condition (either HO or S) was not significant for the ellipse sway area and the CoP path length.

Apart from the COP sway area of the right leg with a medium effect, the effect size is small.

**Fig 2:**
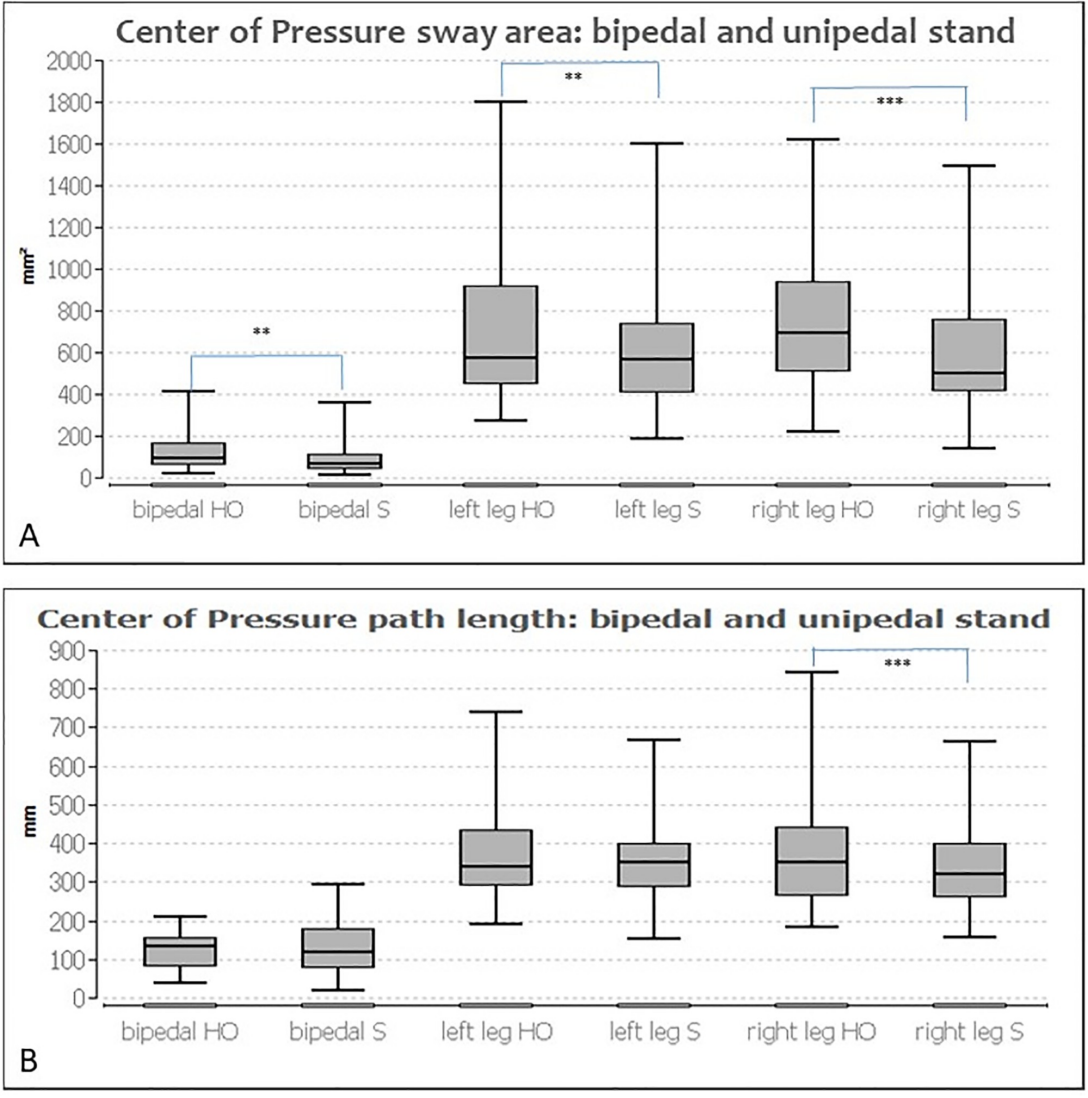
Illustration of A) CoP sway area while bipedal and unipedal standing (left/right) and B) CoP path length while bipedal and unipedal standing (left/right), for both habitual occlusion (HO) and wearing a splint (S). The graphs show box plots with frames representing the 1st and 3rd quartiles and the median demonstrating the thick bar within this box. The upper and lower whiskers illustrate the minimum and maximum.*: p ≤ 0.05, **: p ≤ 0.01, ***: p ≤ 0.001.

Fig 2 shows the ellipse sway area and path length for both measurement conditions in comparison for the bipedal stance and unipedal stance.

The descriptive comparison of all the evaluation parameters between the left- and right-handers showed no noticeable differences. The comparison of participants with a left-playing leg versus men with a right one also revealed no significant differences after Bonferroni-Holm correction.

## Discussion

The aim of the present study was to determine whether using the application of a splint in an idealized, condylar position close to the centric position can induce changes in cervical mobility and postural stability compared to the habitual occlusion of recreational active males.

In terms of cervical spine mobility, wearing the splint increased minimally rotation to the left and right and lateral flexion to the left and right, but did not affect the flexion and extension of the cervical spine in the sagittal plane; therefore, hypothesis 1 can be verified. With regard to postural stability, wearing the splint reduced the area of sway by approximately 30mm^2^ in the bipedal stance and by approximately 14mm^2^ (left) and 196mm^2^ (right) in the unipedal stance. The CoP path length, i.e., the length of the body’s anterior-posterior deflection in the sagittal plane, also decreased by approximately 31mm in the right unipedal stance. Accordingly, the bipedal sway area of the male participants was especially reduced by wearing the splint. The anterior-posterior sway path length is demonstrably shorter in the right one-legged stance than that of the left. Since only a significant reduction was observed in the right one-legged stance and not in the left, hypothesis 2 has to be falsified, while hypothesis 3, on the other hand, must be verified since no significant differences were recorded in the bipedal stance.

Within this study, wearing a splint had a greater influence on the CoP measurements, especially for the sway areas, compared to the ROM measurements. Some theories state [15, 16, 19] that bringing the jaw into a physiologically relaxed centric position relaxes not only the muscles directly around the joint but also leads to a relaxation of the whole neuromuscular system through polysynaptic neuromuscular interactions.

Since the ROM only increased by approximately 0–3°, this can only be regarded as a trend (most effect sizes are small), on average, across all participants, although the individual variance should be taken into account in practice. The reductions in the sway area in the bipedal stance and in the right single-leg stance are more meaningful in the measurement results. In this context, it must be taken into account that the maximum movement executions (ROM) of the cervical spine represent extreme positions, while the sway area provides information on the fluctuation around the habitual neutral standing position. It is usually easier to change the neutral position than the final position of a movement execution (ROM), e.g., due to functional-anatomical structures. Taking this aspect into account, although the differences in maximum movement executions in the cervical spine are only a trend, they are, nevertheless, an indicator that small changes are also possible here. Furthermore, whole-body movements are often initiated by movements of the head. If these can be minimally increased in their maximum range of motion, this may yield positive effects on the overall movement execution.

Why the sway area is reduced more than 10 fold compared to the left single-leg stance and why the CoP path length is minimally reduced only when standing on the right leg should be analyzed in further studies, among other things. Based on the information provided by the participants regarding their dominant arm and leg sides and the comparisons made accordingly, an influence of these factors can be excluded. Moreover, despite the fact that the comparison between the left and right dominant leg did not reveal any significant differences, the results demonstrate that in the single-leg stance there are significances only to the right side. However, this fact needs to be analyzed more thoroughly in further studies. Besides, despite the fact that the comparison between the left and right dominant leg did not reveal any significant differences in the HO and S conditions, the results demonstrate that in the single-leg stance, when comparing the two occlusion conditions, there are significances only to the right side. For whatever reason, there was an interaction on the right side of the body between the occlusion conditions of the splint vs. no splint, while on the left side the sway area remained almost the same, needs to be investigated in further analyses. The present study favors the theory of dependency of both body stability and range of motion of the cervical spine on the mandibular position and CMS, respectively. The reason why the results of Ferrario et al. [38], Perinetti et al. [40] and Michelotti et al. [39] on postural control and cervical spine mobility differ from the present results might be due to the fact that these studies investigated different provoked dental occlusion position i.e. via symmetrical/asymmetrical placed cotton rolls instead of a custom made mandibular split in a position close to the centric relation. Additionally, in these studies no athletes were studied and the testing was gender-unspecific.

Analogous to the results of the present study is the conclusion of the review by Cesanelli [76], in which, among other things, occlusal splint characteristics and occlusion experimental conditions or exercise biomechanics were analyzed. They suggested the application of the occlusal splints as a way to improve either athletes’ or individuals’ oral health and additionally as a potential tool optimizing marginal aspects of exercise performance. Cardoso et al. [77] also proved that a jaw-protruding splint can have a positive biophysical effect on running-performance-related parameters. Here, triathletes showed an approx. 4% reduction in contact time and stride length as well as an approx. 4% increase in stride frequency by wearing the forwarding splints. However, jaw repositioning splints showed no influence at different running intensities [76].

In addition, sport-specific analyses should be performed in the future since Militi et al. [78] suggested that, depending on the sport, a changed mandibular position could have different effects.

Furthermore, it is important in the future to examine athletes who already suffer from temporomandibular dysfunction. The extent to which the effects achieved in these future examinations differ from the present results must be investigated in further studies. The overriding question of why the nearby structures experience less improvement than the structures located much further caudally also requires further research. It is also unclear whether these positive effects from wearing the splint have a direct impact on the performance of the athletes.

When interpreting the data and placing them in the scientific context, it is important to note that no prefabricated splint was used in the present study, but rather a splint made individually for each participant in physiological relation, close to the centric. Therefore, on the one hand, due to divergent manufacturing processes used for producing commercially available splints and, on the other hand, the processes used in producing custom-made splints in relation to ready-made splints, the present results are difficult to compare with other studies [54–56]. This is because the gripping of the position of the maxilla in relation to the skull plays just as important a role here as the centric does in taking of the bite. However, despite divergent splint fabrication methods, the present results confirm the statements of previous studies describing a functional correlation or dependence between posture and the TMS [46, 79–81].

The positioning of the temporomandibular joint by means of a custom-made splint seems to have a slightly positive influence on the immediately adjacent system of the cervical spine. Certainly, the direct anatomical proximity of the interacting structures between the temporo-mandibular joint and the cervical spine is decisive here, as well as the neural connection of afferent and efferent nerve fibers in addition to the muscular and fascial connection of the individual body segments [82]. Lee et al. [82] stated the hypothesis that afferent nerve fibers of the TMS are directly interconnected with the efferent neurons that influence posture and body stability; it is this direct neural circuitry that could promote the "ad hoc effect". Moreover, in previous studies, different authors have reported the rapid, reflexive responses of body posture to changes in the mandibular position [83–85]. Furthermore, it is assumed that the CMS is interconnected with the neuromuscular system of all body structures via the central nervous system [86].

The study also has limitations, which are indicated in the following. The present findings are the results of an "ad hoc effect" that occurred in the context of this cross-sectional analysis as the recreational athletes wore the splint for 20 minutes for the first time before the measurement. To what extent these improvements now manifest themselves in the longer term and whether they are still detectable to the same extent after 6 or 12 months cannot be concluded from this study and would be desirable to investigate in future studies. Furthermore, only men were measured, so that the effect of women, unathletic people or patients with TMD is missing using the same study design. A further limitation is that the measurements are only conducted in standing or sitting position and under dynamic conditions such as the favored sporting activity.

However, increases in the range of motion in the cervical spine and improvements in stance stability indicate a positive influence on intra- and intermuscular coordination during movement, which may then be reflected in athletic movements.

However, the limitations are also contrasted by some strengths. This study was conducted on a homogeneous group of subjects, namely subjectively healthy male recreational athletes without TMD. In addition, centric bite registration was always performed by only one dentist. Furthermore, it should be taken into account that two measurement systems were used, since both the range of motion (ROM) of the cervical spine and postural stability are important for an economical and motor adequate adaptation of the body to any situation. In addition, all splints are fabricated by one practitioner using the same procedure, so there is no practitioner-specific variance to consider here. As a clinical application, it can be concluded that wearing the splint allows small biophysical changes to be measured in terms of the ROM of the cervical spine and balance stability. Whole-body movements are often initiated by movements of the head. If these can be minimally increased in their maximum range of motion, this may yield positive effects on the overall movement execution. By improving stance stability, the athlete can perform more precise and targeted movements. With better stability and less body sway, the body has to activate fewer compensation and balancing mechanisms and can therefore focus more effectively on the exercise itself. However, the extent of the change is always divergent, which is due to a different physical condition (among other things, injuries, complaints, muscular development).

## Conclusion

It can be summarized that by means of a splint that holds the temporomandibular joint close to the centric jaw relation, the cervical spine mobility tends to increase in its bilateral head rotation and bilateral lateral flexion, while the stance stability improves in the bipedal and right unipedal stances. These conditions may form a better baseline for performing athletic activities. However, these results are an ad hoc effect and, thus, conclusions concerning long-term effects cannot be drawn.
